# Fabrication of (SiC-AlN)/ZrB_2_ Composite with Nano-Micron Hybrid Microstructure via PCS-Derived Ceramics Route

**DOI:** 10.3390/ma14020334

**Published:** 2021-01-11

**Authors:** Aidong Xia, Jie Yin, Xiao Chen, Zhengren Huang, Xuejian Liu, Wei Liu

**Affiliations:** 1 State Key Laboratory of High Performance Ceramics and Superfine Microstructures, Shanghai Institute of Ceramics, Chinese Academy of Sciences, Shanghai 200050, China; xiaaidong@student.sic.ac.cn (A.X.); chenxiao@student.sic.ac.cn (X.C.); xjliu@mail.sic.ac.cn (X.L.); 2 College of Materials Science and Opto-Electronic Technology, University of Chinese Academy of Sciences, Beijing 100049, China; 3 Ningbo Institute of Materials Technology and Engineering, Chinese Academy of Sciences, Ningbo 315201, China; 4 School of Mechatronic Engineering, Guangdong Polytechnic Normal University, Guangzhou 510635, China; liuwei@gdut.edu.cn; 5 School of Electromechanical Engineering, Guangdong University of Technology, Guangzhou 510006, China

**Keywords:** (SiC-AlN)/ZrB_2_ composite, polymer-derived ceramics, nano-micron hybrid microstructure, hot pressing, mechanical properties

## Abstract

In this work, a (SiC-AlN)/ZrB_2_ composite with outstanding mechanical properties was prepared by using polymer-derived ceramics (PDCs) and hot-pressing technique. Flexural strength reached up to 460 ± 41 MPa, while AlN and ZrB_2_ contents were 10 wt%, and 15 wt%, respectively, under a hot-pressing temperature of 2000 °C. XRD pattern-evidenced SiC generated by pyrolysis of polycarbosilane (PCS) was mainly composed by 2H-SiC and 4H-SiC, both belonging to α-SiC. Micron-level ZrB_2_ secondary phase was observed inside the (SiC-AlN)/ZrB_2_ composite, while the mean grain size (MGS) of SiC-AlN matrix was approximately 97 nm. This unique nano-micron hybrid microstructure enhanced the mechanical properties. The present investigation provided a feasible tactic for strengthening ceramics from PDCs raw materials.

## 1. Introduction

With a representative feature of strong covalent bonding, SiC possesses high strength, hardness, and chemical stability, as well as excellent thermal shock and wear resistance. Its products mainly include functional ceramics, abrasives, and high temperature-resistant materials with wide applications [1,2]. Polymer-derived ceramics (PDCs) technology receives increasing attention on the fabrication of SiC ceramics [3,4,5]. Different from the traditional ceramic preparation process, the PDCs technology is to crosslink organic polymers, called “ceramic precursors” (for example, polysiloxane, polycarbosilane, polysilazane), before pyrolyzing at high temperatures, releasing CO, CO_2_, H_2_, CH_4_, HCHO, and other small-molecule gases, leaving the ceramic skeleton, finally completing the transformation from polymers to ceramics [6,7,8]. The most commonly used ceramic precursor for preparing SiC is polycarbosilane (PCS).

From a microscopic point of view, no matter which ceramic precursor is used, the microstructure of PDCs is similar. PDCs are mainly composed of a network by free carbon and Si-rich nanodomains inside [9], while grains of PDCs are usually nanosized. This unique microstructure renders excellent physical and chemical properties in PDCs [10,11], such as thermal [12,13,14,15], electrical [16,17,18], electrochemical energy storage [19,20], and electromagnetic properties [21,22,23,24,25]. Nevertheless, during the pyrolysis of the ceramic precursors, a large amount of small molecule gas would be released, and the green body would shrink, with the formation of pores and cracks. Defects, such as pores and cracks, could deteriorate the mechanical properties of PDCs, thus limiting the application of PDCs [5]. For example, Huang et al. [26] used PCS as the ceramic precursor to prepare lightweight SiC by freeze-casting with a porous structure, and its flexural strength was only 3.7–11.3 MPa. The flexural strength of SiC foam prepared by Durif et al. [27] using boron-modified PCS was only 3.49 ± 0.59 MPa. At present, there are two general methods to improve the mechanical properties of PDCs. One is to introduce a reinforcing phase. The flexural strength of C/ZrC/SiC composite prepared by Chen et al. [28] through the PCS-derived ceramics route can reach 319 MPa. Laadoua et al. [29] used allylhydrido-PCS (SMP-10) as the ceramic precursor to prepare ZrC/SiC composites with a Young’s modulus of 402 GPa through a spark plasma sintering (SPS) process. The other is to use densification processes, such as polymer impregnation pyrolysis (PIP) and chemical vapor infiltration (CVI). He et al. [30] used PCS as the ceramic precursor to increase the flexural strength of SiC prepared by stereolithography from 15 MPa to 205 MPa using the PIP process. Xiong et al. [31] used the CVI process to increase the tensile strength of the 3D printed PCS-derived SiC lattice from 3.3 MPa to 47.3 MPa. Yet PIP and CVI processes are complicated, time-consuming. Compared with PIP and CVI, hot-pressing technique can obtain densified components with fine grains and outstanding mechanical properties.

In this study, (SiC-AlN)/ZrB_2_ composite prepared from PCS precursors and hot-pressing were investigated. It exhibited outstanding mechanical properties with three-point flexural strength of up to 460 ± 41 MPa. AlN and α-SiC belong to the same crystal structure with similar lattice parameters. They could form a solid solution by wide compositional and temperature ranges. In the low-AlN-content region, the grain growth of SiC was curbed by AlN, which can strongly enhance its strength [32,33]. Belonging to an ultra-high temperature ceramic family, ZrB_2_ shares the same hexagonal system as AlN, which could potentially increase the high temperature service behavior [34,35].

## 2. Materials and Methods

### 2.1. Raw Materials

Liquid polycarbosilane (PCS, Institute of Chemistry, Chinese Academy of Sciences, Beijing, China) was used as the precursor. Dicumyl peroxide (DCP, Institute of Chemistry, Chinese Academy of Sciences, Beijing, China) was used as a crosslinking agent for liquid PCS. AlN (∼0.1 μm) powder was obtained from Tokuyama Co. (Tokyo, Japan). ZrB_2_ (~1 μm) powder was obtained from Shanghai Buwei Applied Materials Technology Co., Ltd. (Shanghai, China). All chemicals were used without further purification.

### 2.2. Processing

#### 2.2.1. Crosslink

An amount of 0.4 wt% DCP was added to the liquid PCS and ultrasonically dispersed for 10 min. Then, the mixed liquid was poured into an alumina porcelain boat and was put in a tube furnace, crosslinked at 140 °C for 4 h under N_2_ atmosphere to obtain crosslinked PCS, and ground it into powder in an agate mortar.

#### 2.2.2. Preparation of Composite Materials

The crosslinked PCS, ZrB_2_, AlN, and SiC balls (as grinding media) were mixed with ethanol in a polypropylene tank. The recipe of starting compositions was listed in Table 1. Then, a planetary ball mill was used to mill the mixture at 300 r min^−1^ for 4 h. After grinding, the slurry was dried at 60 °C for 12 h and then sieved. The obtained powder was heated to 1200 °C at a rate of 1.5 °C min^−1^ in Ar atmosphere and kept for 0.5 h for the PCS pyrolysis. The mixed powder obtained after pyrolysis was sieved again and poured into a 40 × 40 mm^2^ graphite mold. The hot-press sintering was carried out at 1950–2100 °C under Ar atmosphere for 1 h to increase the density. P70Z15A15 was selected as the initial sample to explore the effect of temperature on performance. Then, the effect of AlN content on performance was explored under the most suitable temperature.

### 2.3. Characterization

The relative density of the prepared (SiC-AlN)/ZrB_2_ composite was measured by Archimedes method, each sample was tested at least 3 times, and the theoretical density was determined by the mixing rule. The samples were processed into 3 × 4 × 36 mm^3^ test strips, and then the flexural strength and elastic modulus were measured by the bending test. All bending tests were performed on the same mechanical tester (Instron-119, Instron, Boston, MA, USA) with a three-point bending method at a crosshead speed of 0.5 mm min^−1^ and a fulcrum span of 30 mm. The diameter of rollers is 4.5 mm, the testing temperature is 25 °C, and the relative humidity is 50%. At least 5 test strips must be measured for each sample. The strain *D* and the applied load *P* were recorded by the machine. The corrected strain *D*_0_ was measured by a mechanical gauge under the applied load *P*_0_ (the maximum value of all recorded loads). The elastic modulus *E* can be calculated according to Formula (1):

(1) $$E=\frac{L^{3}}{4BH^{3}\left(\frac{D}{P}-\frac{D_{0}}{P_{0}}\right)}$$

where *L* is the fulcrum span, *B* and *H* are the width and height of the test strip, respectively. The polishing machine (UNIPOL-802, SHENYANG KEJING, Shenyang, China) was used to polish the samples to 0.5 μm. A microhardness tester (Willson-Wolpert 2100B, Instron, Boston, MA, USA) was used to measure the hardness of the polished samples under a 5 N Vickers pyramid diamond indentation load with a dwell time of 10 s. At least 5 points must be measured for each sample.

A thermal analysis mass spectrometer (STA449C, NETZSCH, Selb, Germany) was used to characterize the thermogravimetric (TG) curve of the crosslinked PCS. The crosslinked PCS was heated to 1400 °C at a rate of 10 °C/min in Ar atmosphere. The TG curve was tested once. X-ray diffractometer (XRD, CuKα radiation, 40 KV, 40 mA, D8Advance, Bruker, Karlsruhe, Germany) was used to characterize the phase of the prepared (SiC-AlN)/ZrB_2_ composite, the scanning step was 0.02°/step, the scanning rate was 0.3 s/step, and the scanning range was 20°–80°. XRD was tested once on samples prepared at 1950, 2000, 2050, and 2100 °C, respectively. The microstructure of the prepared (SiC-AlN)/ZrB_2_ composite was observed by scanning electron microscope (SEM, Verios G4, FEI, Hillsboto, OR, USA) and transmission electron microscope (TEM, JEM-2100F, JEOL, Tokyo, Japan) equipped with energy dispersive spectrometer (EDS, AMETEK EDAX, Berwyn, PA, USA). The energy resolution of EDS is 150 eV. The mean grain size (MGS) is calculated by randomly selecting 50 grains, measuring the maximum diameter, and then taking the average value.

## 3. Results and Discussion

**Figure 1:**
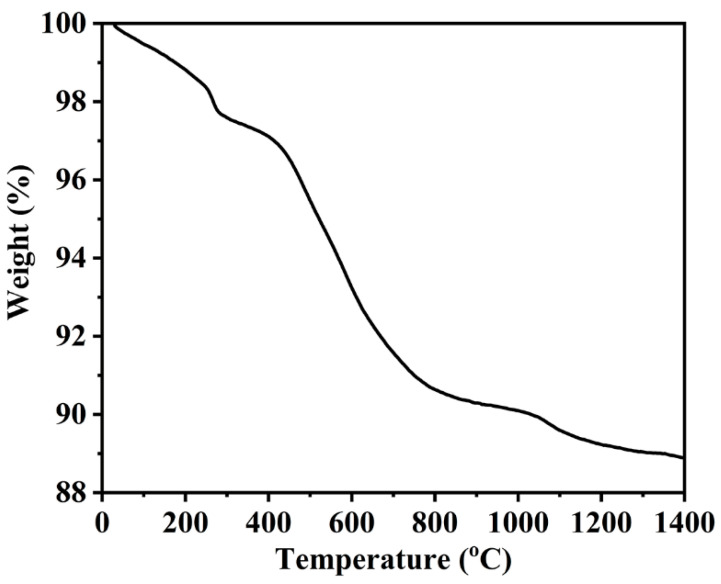
Thermogravimetric (TG) curve of crosslinked polycarbosilane (PCS).

Raw PCS used in our study was in liquid state. In order to increase the ceramic yield, crosslink process was conducted. Figure 1 shows the thermogravimetric (TG) curve of as-crosslinked PCS. The TG curve had a steep slope in the range of 240–290 °C. Because PCS had stronger dehydrogenation coupling in this temperature range, and the gas produced was larger. The TG curve had a larger slope in the range of 450–800 °C. PCS experienced a violent pyrolysis reaction, released a large proportion of small-molecule gas, which was the main temperature range for PCS pyrolysis, so the mass loss in this range was relatively high. The mass loss of PCS was terminated after 1200 °C, indicating that by this temperature, ceramic skeleton was left behind and the transformation from polymer to ceramic was completed. The ceramic yield of PCS was 89%.

**Figure 2:**
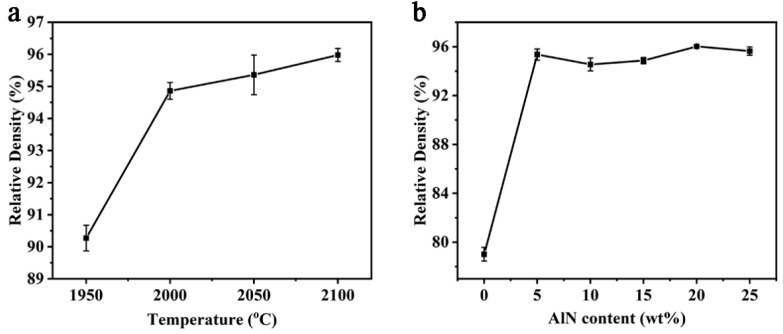
Relative density: (**a**) P70Z15A15 at different sintering temperatures and (**b**) samples with 15 wt% ZrB_2_ sintered under 2000 °C by different AlN content.

The relative density of the P70Z15A15 at different sintering temperatures was shown in Figure 2a. When the temperature was 1950 °C, the relative density of the sample was 90.27%. As the temperature increased, the relative density increased rapidly. When the temperature increased to 2000 °C, porosity decreased, and the relative density increased (94.86%). As the temperature continued to increase, the relative density further increased (yet slowly), eventually reaching 95.98% by 2100 °C. The relative density of samples with 15 wt% ZrB_2_ sintered under 2000 °C by different AlN content was shown in Figure 2b. Without the AlN addition, the relative density was only 79.01%. The relative density increased quickly by the introduction of a small amount of AlN: it increased to 95.36% by merely adding 5 wt% AlN as sintering aid [36]: the formation of solid solution was beneficial to reduce the grain boundary energy of SiC and AlN, enhanced the diffusion of Si, C, Al, and N atoms in the solid solution lattice, and hence improved the sinter-ability [37]. The result proved that the addition of 5 wt% AlN could efficiently promote densification progress.

**Figure 3:**
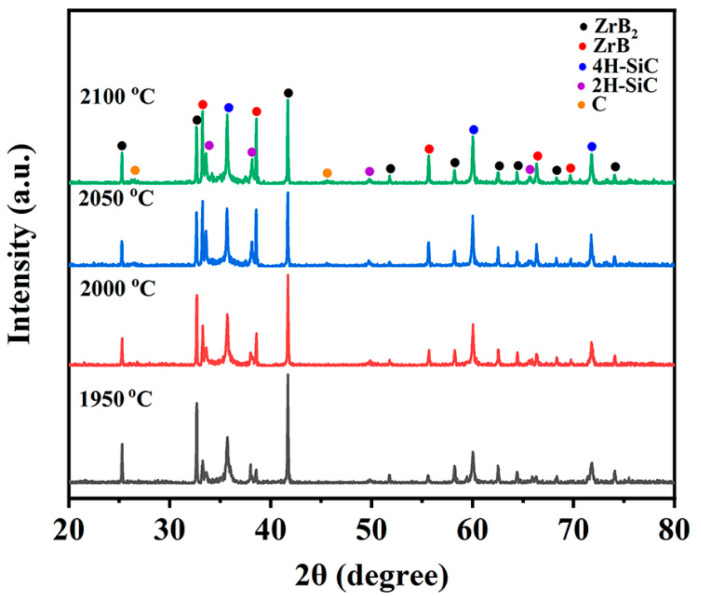
XRD patterns of P70Z15A15 by the variation of sintering temperatures.

The XRD patterns of the P70Z15A15 by different temperatures were shown in Figure 3. ZrB_2_, ZrB, C, and SiC peaks were mainly detected, without the presences of AlN peaks. The reason is as follows: AlN dissolves into the SiC lattice to form a continuous solid solution in the present processing temperature. 2H-SiC and 4H-SiC were observed, both belonging to α-SiC. This was also in agreement with the SiC-AlN phase diagram [38]. During the pyrolysis process, PCS will not only release small-molecule gas, but also a small amount of free carbon will inevitably be generated, so the C peak will be detected in XRD patterns [1]. As the temperature increased, the intensity of ZrB peak gradually increased, which indicated that higher temperature was beneficial to the generation and stability of ZrB. Further studies are under way on investigating ZrB.

**Figure 4:**
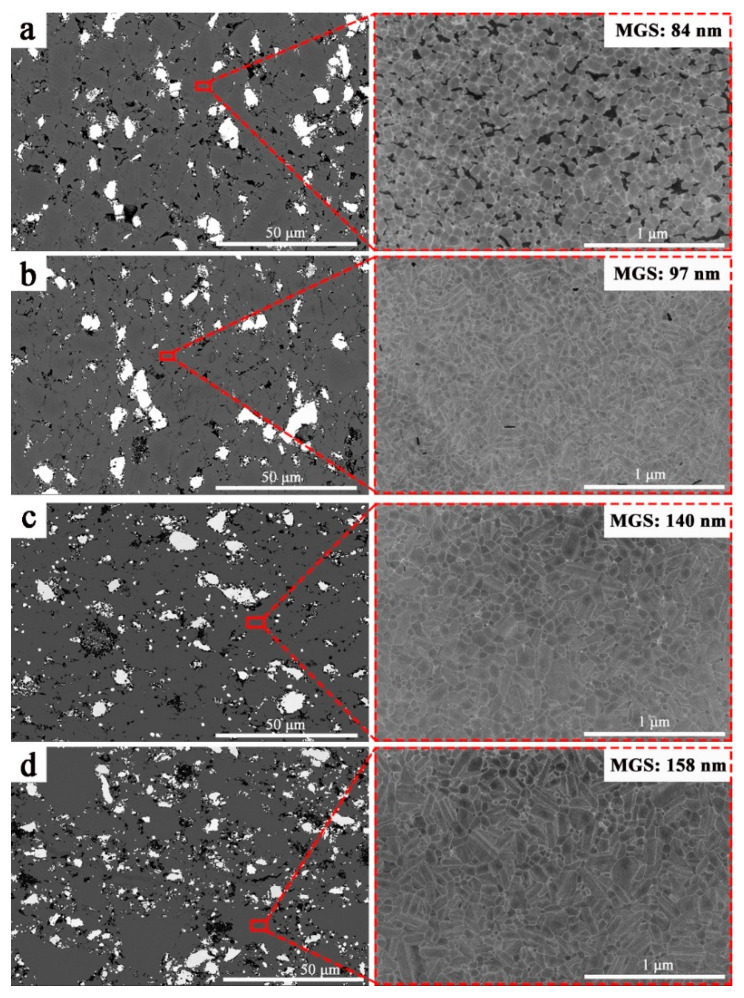
Scanning electron microscope (SEM) images of the polished surface after ion etching at different temperatures: 1950 °C (**a**); 2000 °C (**b**); 2050 °C (**c**); 2100 °C (**d**).

The SEM images of the P70Z15A15 at different temperatures were shown in Figure 4. The white phase was ZrB_2_. The overall distribution of ZrB_2_ was uniform, but agglomeration phenomenon was also observed between ZrB_2_ particles. As a reinforcing phase, ZrB_2_ can increase the crack propagation pathway, hinder the crack propagation, cause crack deflection, detour, or branch, thereby improving the mechanical properties [34]. The black areas were pores. Due to ion etching on the sample surface, an etching boundary was formed in areas with more pores. The gray area was the solid solution matrix by SiC-AlN. It can be seen from Figure 4a that when the temperature was 1950 °C, the grain size distribution of the SiC-AlN solid solution became more uniform, its mean grain size (MGS) was 84 nm, and the shape was basically equiaxed. Since the sample was not densified under this temperature, more pores appeared. When the temperature increased further up to 2000 °C, oriented grain growth occurred, and elongated grains were formed (Figure 4b). The MGS was 97 nm. Such presence of nano-scale grains had a strong influence in enhancing the mechanical properties of the (SiC-AlN)/ZrB_2_ composite [39]. When the temperature further increased, the elongated crystal grains further increased, and the MGS also increased with the temperature increase (Figure 4c,d). At 2100 °C, the MGS was around 158 nm. The increase in grain size would play a negative role in mechanical properties of the (SiC-AlN)/ZrB_2_ composite. On the whole, secondary ZrB_2_ phase in the (SiC-AlN)/ZrB_2_ was on micron-level, while (SiC-AlN) was on nano-level. This nano-micron composite structure was a critical reason for revealing mechanical properties of (SiC-AlN)/ZrB_2_ composite.

**Figure 5:**
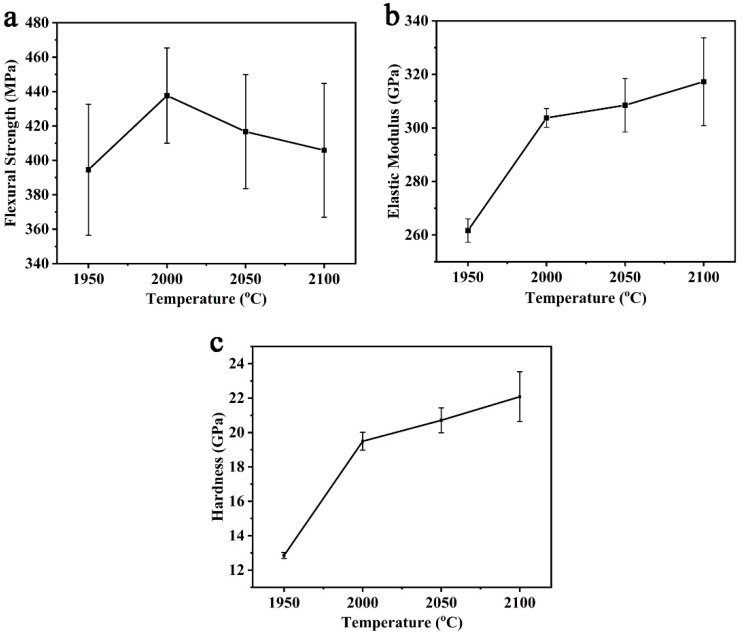
Mechanical properties of P70Z15A15 at different temperatures: flexural strength (**a**); elastic modulus (**b**); Vickers hardness (**c**).

The mechanical properties of the P70Z15A15 by different sintering temperatures were shown in Figure 5. It can be seen from Figure 5a that the flexural strength of (SiC-AlN)/ZrB_2_ increased by increasing temperature in the range of 1950–2000 °C, reaching the maximum value (438 ± 28 MPa) at 2000 °C. The increase in flexural strength from 1950 °C to 2000 °C was due to the removal of residual pores. In this temperature range, the relative density of (SiC-AlN)/ZrB_2_ increased from 90.27% to 94.86% (Figure 2a). The increase in relative density greatly increased the strength. However, from 2000 °C to 2100 °C, the flexural strength of (SiC-AlN)/ZrB_2_ composite showed a slight decrease by 7.26%. When the temperature further increased from 2000 °C to 2100 °C, the relative density increased from 94.86% to 95.98% (Figure 2a). However, as the temperature increased, the (SiC-AlN)/ZrB_2_ composite experienced over-sintering, and obvious grain growth occurred (increased by 60%, Figure 4), which has a stronger impact on its flexural strength. According to the Hall–Petch relationship, the flexural strength is inversely proportional to grain size [40]. Therefore, compared with the increase in relative density, grain growth effect dominated in the range of 2000–2100 °C. It can also be seen from Figure 5b,c that elastic modulus and hardness followed the same regularity with relative density (Figure 2a). As the temperature increased from 1950–2000 °C, elastic modulus and hardness increased remarkably (by 16.08% and 51.67%, respectively) due to the increase in relative density. However, from 2000 °C to 2100 °C, the relative density increased only by 1.12%, and the corresponding elastic modulus and hardness increase (by 4.45% and 13.29%, respectively) was presumably due to the solid solutioning effect. The elastic modulus and hardness were 317 ± 16 GPa and 22.08 ± 1.45 GPa, respectively, for P70Z15A15 sintered under 2100 °C.

**Figure 6:**
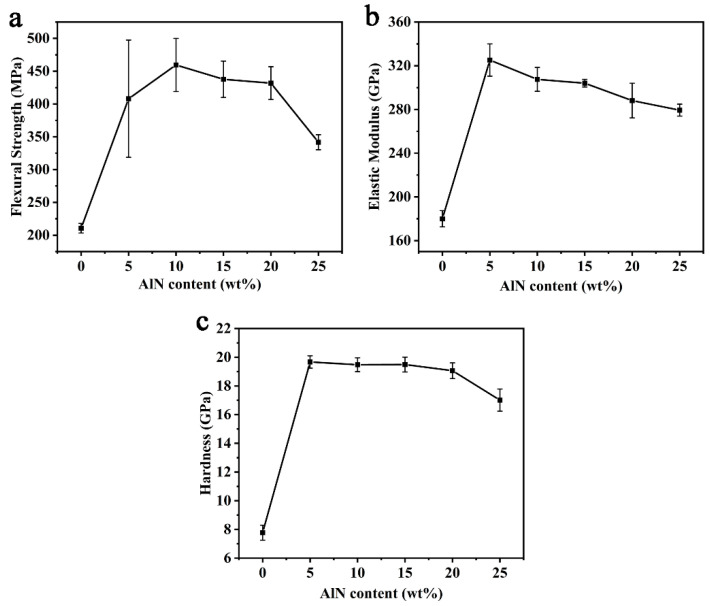
Mechanical properties of samples by the addition of 15 wt% ZrB_2_ at 2000 °C under different AlN content: flexural strength (**a**); elastic modulus (**b**); Vickers hardness (**c**).

Figure 6 shows the mechanical properties of (SiC-AlN)/ZrB_2_ composite by the addition of 15 wt% ZrB_2_ at 2000 °C under different AlN content. Under different AlN content, the strength of (SiC-AlN)/ZrB_2_ increased firstly before decreasing (Figure 6a). The flexural strength was only 211 ± 7 MPa without AlN addition. Additionally, when the AlN content was 10 wt%, the flexural strength of (SiC-AlN)/ZrB_2_ composite increased up to 460 ± 41 MPa (increased by 118% compared to counterpart without AlN). The elastic modulus (Figure 6b) and Vickers hardness (Figure 6c) of (SiC-AlN)/ZrB_2_ composite increased firstly and then decreased as well, both of which reached maximum values with 5 wt% AlN: 325 ± 15 GPa (1.8 times that of the sample without AlN) for elastic modulus, and 19.67 ± 0.43 GPa (2.53 times that of the sample without AlN) for hardness, respectively. Such remarkable increases were mainly due to the relative density increase. Meanwhile, the MGS of (SiC-AlN)/ZrB_2_ composite was 97 nm (Figure 4b). This was due to the in-situ formation of SiC by precursor pyrolysis and the effect of AlN on refining SiC grains. Under the sintering temperature range of SiC, AlN has a higher vapor pressure and a higher diffusion coefficient than SiC. The migration rate of AlN to SiC is much greater than its reverse migration rate. So that AlN can preferentially diffuse to the surface of SiC grains. On one hand, it provided a diffusion barrier and inhibited the grain growth of SiC. On the other hand, AlN can react with SiC in a solid solution, and re-nucleate around the mutual-interface, and refine the grains [41]. The grain refinement down to nanoscale decreased the critical defect size and improved the material strength. It is generally believed that the effect of AlN on refining SiC grains mainly occurs in the low AlN range. Meanwhile, AlN is relatively “softer” (~12 GPa) than SiC (~20 GPa) [42]. Therefore, the addition of higher AlN content (>10 wt%) could hardly improve mechanical properties of (SiC-AlN)/ZrB_2_ composite to higher values.

**Figure 7:**
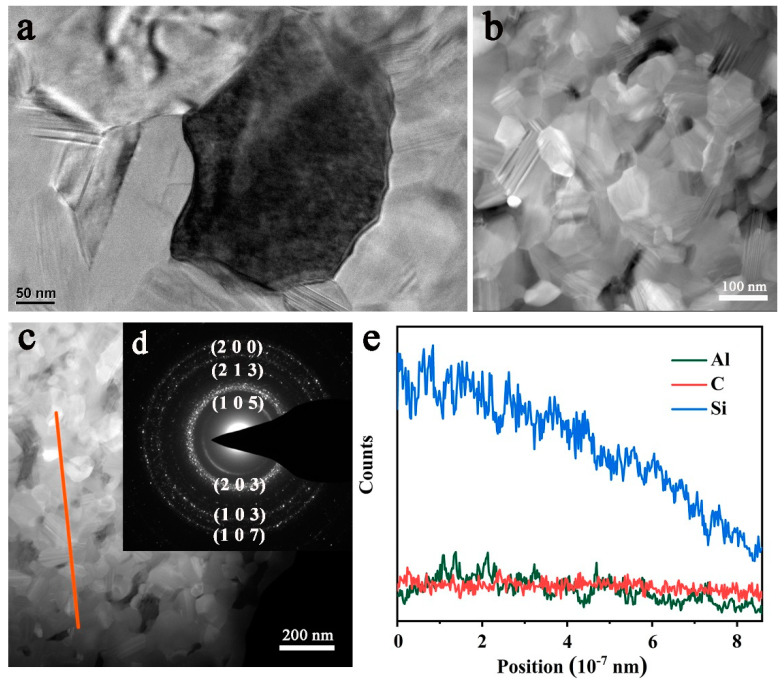
(**a**)Transmission electron microscope (TEM); (**b**,**c**) high-angle annular dark field (HAADF); (**d**) selected area electron diffraction (SAED); (**e**) line scan of the orange line in (**c**) in HAADF-STEM mode.

Figure 7a shows the transmission electron microscope (TEM) image of the P75Z15A10 at 2000 °C. ZrB_2_ (dark phase), and SiC-AlN solid solution (gray phase) were observed. Figure 7b,c analyzed the SiC-AlN solid solution by high-angle annular dark field (HAADF) technique. It can be seen from Figure 7b that the grain size of the SiC-AlN solid solution phase was 100 nm, and the morphology of both equiaxed and elongated grains was consistent with SEM observation (Figure 4b). Meanwhile, wrinkles were observed inside the grains, which were caused by phase segregation. Figure 7e was the line scanning of (orange line) in Figure 7c. The elemental distribution of carbon was uniform. Compared with C, slight fluctuation in compositional distribution of Al was observed. Gradient segregation of Si was more serious (Figure 7c), indicating that the solid solution formation of SiC-AlN might not accomplished. Figure 7d was the selected area electron diffraction (SAED) pattern of the SiC-AlN solid solution phase. The diffraction pattern of nano matrix phase was a polycrystalline ring, the calculated lattice plane from the inside to the outside was (1 0 5), (2 0 3), (2 1 3), (1 0 3), (2 0 0), (1 0 7). Among them, (1 0 5), (2 0 3), and (1 0 7) crystal planes belong to 4H-SiC, and (2 1 3), (1 0 3), and (200) planes belong to 2H-SiC, which was consistent with XRD verifications.

## 4. Conclusions

High-performance (SiC-AlN)/ZrB_2_ composite fabricated by hot-pressing from PCS precursors. The composite had outstanding mechanical properties. When the AlN and ZrB_2_ contents were 10 wt% and 15 wt%, and the hot-pressing temperature was 2000 °C, its flexural strength reached 460 ± 41 MPa. Compared with other PDCs materials mentioned in the literature, the mechanical properties of this composite are in a leading position. The excellent mechanical properties of (SiC-AlN)/ZrB_2_ composite can be attributed mainly to several reasons: pyrolyzing PCS, which generated nano-sized SiC in situ; the addition of AlN, on the one hand, can promote the sintering of multiphase ceramics, on the other hand, it can form a continuous solid solution with SiC to refine the SiC grains; the use of hot-pressing allows heating and pressurization to be carried out at the same time, which makes up for the shortcomings of PDCs that are prone to defects such as pores and cracks; the MGS of secondary ZrB_2_ was 1 μm, while the MGS of the SiC-AlN solid solution phase was 97 nm. This unique nano-micron hybrid structure further improved the mechanical properties. The successful preparation of this composite provides ideas for solving the problem of insufficient mechanical properties of PDCs, and it is also hopeful that it will be widely used in the preparation of sensors, membranes, coatings, and components for usage under harsh environments. In recent years, functionalization of PDCs is also a hot topic in major international conferences. Therefore, PDCs and their composites will show very broad prospects in the near future.

## Figures and Tables

**Table 1:** Starting compositions of different samples.

Label	PCS (wt%)	ZrB_2_ (wt%)	AlN (wt%)
P85Z15A0	85	15	0
P80Z15A5	80	15	5
P75Z15A10	75	15	10
P70Z15A15	70	15	15
P65Z15A20	65	15	20
P60Z15A25	60	15	25

## Data Availability

Data sharing not applicable.
